# Strained GaAs/InGaAs Core-Shell Nanowires for Photovoltaic Applications

**DOI:** 10.1186/s11671-016-1384-y

**Published:** 2016-04-01

**Authors:** K. Moratis, S. L. Tan, S. Germanis, C. Katsidis, M. Androulidaki, K. Tsagaraki, Z. Hatzopoulos, F. Donatini, J. Cibert, Y. -M. Niquet, H. Mariette, N. T. Pelekanos

**Affiliations:** Department of Materials Science and Technology, University of Crete, P.O. Box 2208, 70013 Heraklion, Greece; CEA, INAC, 17 rue des Martyrs, 38054 Grenoble cedex 9, France; Université Grenoble Alpes, F-38000 Grenoble, France; Microelectronics Research Group, IESL-FORTH, P.O. Box 1385, 71110 Heraklion, Greece; Department of Physics, University of Crete, P.O. Box 2208, 70013 Heraklion, Greece; CNRS, Institut NEEL, F-38000 Grenoble, France

**Keywords:** Core-shell GaAs/InGaAs nanowires, Nanostructure characterization, Micro-Raman, Photoluminescence, Cathodoluminescence, Micro-photoluminescence

## Abstract

We report on the successful growth of strained core-shell GaAs/InGaAs nanowires on Si (111) substrates by molecular beam epitaxy. The as-grown nanowires have a density in the order of 10^8^ cm^−2^, length between 3 and 3.5 μm, and diameter between 60 and 160 nm, depending on the shell growth duration. By applying a range of characterization techniques, we conclude that the In incorporation in the nanowires is on average significantly smaller than what is nominally expected based on two-dimensional growth calibrations and exhibits a gradient along the nanowire axis. On the other hand, the observation of sharp dot-like emission features in the micro-photoluminescence spectra of single nanowires in the 900–1000-nm spectral range highlights the co-existence of In-rich enclosures with In content locally exceeding 30 %.

## Background

Semiconductor nanowires (NWs) and NW-based heterostructures are currently under extensive research, due to the unique properties and prominent quantum phenomena emerging from them. For instance, there are numerous reports of NW structures containing quantum dots, which can act as efficient sources of single photons or entangled photon pairs [1–5]. Another application area where NW arrays attract wide interest is in next-generation cost-effective and high-efficiency photovoltaic (PV) devices, based on two main reasons: first, their relaxed lattice-matching requirements, due to easy strain accommodation at the NW free surface, providing flexibility in substrate selection and band-gap engineering. Second, the possibility for lesser material utilization, due to enhanced light absorption in NW arrays, based on their inherent anti-reflecting properties [6], increased light trapping within the array [7], and resonant wave-guiding properties [8]. Recently, a single-junction solar cell based on InP NWs [9] with an efficiency *η* ≈ 13.8 % and a single GaAs NW PV device [10] with *η* ≈ 40 % have been reported, showing the real potential of NW solar cells to compete directly with other thin-film solar cell technologies. Another direction worth exploring for NW solar cells is the possibility to utilize piezoelectric (PZ) fields present in strained NW heterostructures, in order to obtain enhanced NW-based PV devices. As first estimated by Boxberg et al. [11], a significant *axial* PZ field of 60–80 kV/cm can develop in InAs/InP core-shell NWs, which can be used for efficient carrier sweeping towards the device electrodes. As we have recently shown in [12], axial PZ fields of the order of 7–8 kV/cm may even exist in core-shell GaAs/AlGaAs NWs, having a very small lattice mismatch of 0.05 %. Such fields are already significant for the operation of a PV device and should be taken into account in any related device work. In this work, we report on the preliminary results of the growth and characterization of strained core-shell GaAs/InGaAs NWs, a NW heterostructure which is expected to exhibit pronounced PZ effects.

## Methods

GaAs/InGaAs core-shell NWs are grown in a VG Semicon V80H III-As solid-source molecular beam epitaxy (MBE) system via the vapor liquid solid (VLS) mechanism on n^+^ Si (111) substrates (manufactured by Siltronix). The native oxide-capped Si substrate is loaded into the MBE growth chamber without prior chemical treatment and is annealed in situ at 650 °C for 10 min in order to create pinholes in the native oxide layer that act as nucleation sites for GaAs NWs. The core-shell configuration is achieved by first growing GaAs NWs (“core”) and then encapsulating the GaAs core by a two-dimensional (2D) epitaxial growth of InGaAs shell. A substrate temperature of 600 °C is used for the growth of the GaAs core NWs. Ga droplets are deposited in situ at a flux of 0.4 ML/s for 20 s to act as catalysts that initiate the VLS growth of the GaAs core NWs. Note that the Ga and As fluxes are quoted in terms of the equivalent 2D growth rates estimated from RHEED oscillation analysis during Ga- and As-limited GaAs growths on GaAs (100) surfaces. Following Ga droplet deposition, the shutter of the Ga Knudsen cell (K-cell) is closed for 20 s to allow Ga to relax on the substrate surface and form droplets of uniform diameter. The growth of the GaAs NWs is performed for 30 min using a Ga flux of 0.4 ML/s and an As/Ga flux ratio of ~2. These growth parameters result in GaAs core NWs with a diameter of ~60 nm, height of ~3 μm, and density of 2 × 10^8^ cm^−2^.

Following the growth of the GaAs core NWs, the InGaAs shell growth is initiated by exposing the sample to As flux for 10 min to remove the Ga droplets, while the As K-cell temperature is raised to increase the As/Ga flux ratio to ~5. The substrate temperature is decreased to 500 °C, which is optimal for 2D InGaAs growth. The InGaAs shell growth duration and In flux are varied for each sample in order to achieve NWs with different shell thicknesses and In to Ga stoichiometry. In total, three sets of samples have been grown in different days. Each set comprises three GaAs/InGaAs core-shell NW samples and one GaAs core NW reference sample. In the first set of samples, the InGaAs shell growth duration is fixed at 40 min, while the In flux is varied to produce nominal In shell contents of 2, 5, and 9.5 %, based on 2D InGaAs thin-film calibrations. In the second and third sets of samples, the In flux is fixed with a beam equivalent pressure of 4.55 × 10^−8^ mbar, corresponding to a nominal In content of 9.5 %, while the InGaAs shell growth duration is varied, as summarized in Table 1. Finally, the InGaAs shell is passivated (from oxidation when exposed to the air) by a 3-min growth of a thin GaAs cap layer, having an estimated thickness of about 3 nm.

**Table 1:** InGaAs shell growth duration and nominal In content of the samples grown for this study

Set of samples	InGaAs shell growth duration (min)	Nominal In content (%)
1	40 for all 3 samples	2, 5, 9.5
2	1, 2, 4	9.5 for all 3 samples
3	5, 10, 20	9.5 for all 3 samples

The surface morphology and the structural characteristics of the NWs are investigated by performing field emission-scanning electron microscopy (FE-SEM) on the as-grown samples. The optical properties of the samples are studied by performing macro-PL measurements using a 325-nm He-Cd Kimmon laser. The macro-PL experimental setup consists of a closed circuit liquid He-cooled cryostat in which the sample is mounted and excited by a laser beam focused using a 10-cm quartz lens. The excitation power used for PL is limited to 0.23 mW because of photo-bleaching effects due to the activation of surface-related non-radiative channels [13], which are especially pronounced in the GaAs core NW reference samples. The PL signal is analyzed by a 0.5-m spectrograph with a 600 gr/mm grating and is recorded by a liquid nitrogen (LN)-cooled charge-coupled device (CCD) camera.

In addition, micro-Raman, cathodoluminescence (CL) and micro-PL measurements have been performed on individual GaAs/InGaAs core-shell NWs dispersed on Si substrate with gold metal grid to assist with the identification and tracking of individual NWs of interest. A Nicolet Almega XR micro-Raman setup is used for the micro-Raman measurements, which consists of a 473-nm diode laser with the beam focused to a spot size of 0.5 μm using a ×100 microscope objective with 1.25 numerical aperture (NA). The Raman spectra are analyzed by a 2400-gr/mm grating and are recorded by a CCD camera. For the micro-PL measurements, the sample (with NWs dispersed on grid) is mounted on a LN flow cryostat and excited using the 750-nm *cw* line of a tunable Ti:Sapphire laser, with the beam focused to a spot size of ~1 μm using a ×40 microscope objective with 0.65 NA. The micro-PL signal is analyzed by a 0.75-m spectrograph utilizing a 600-gr/mm grating and is recorded by a back-thinned LN-cooled CCD camera with high quantum efficiency. CL measurements at 5 K are carried out in a FEI Inspect F50 FE-SEM system, which is equipped with a Gatan cryogenic stage and a custom in-house-made light collection system. The CL mappings are recorded over a ±3-nm spectral window using a Horiba IHR 550 spectrometer equipped with a photomultiplier.

## Results and Discussion

**Fig. 1:**
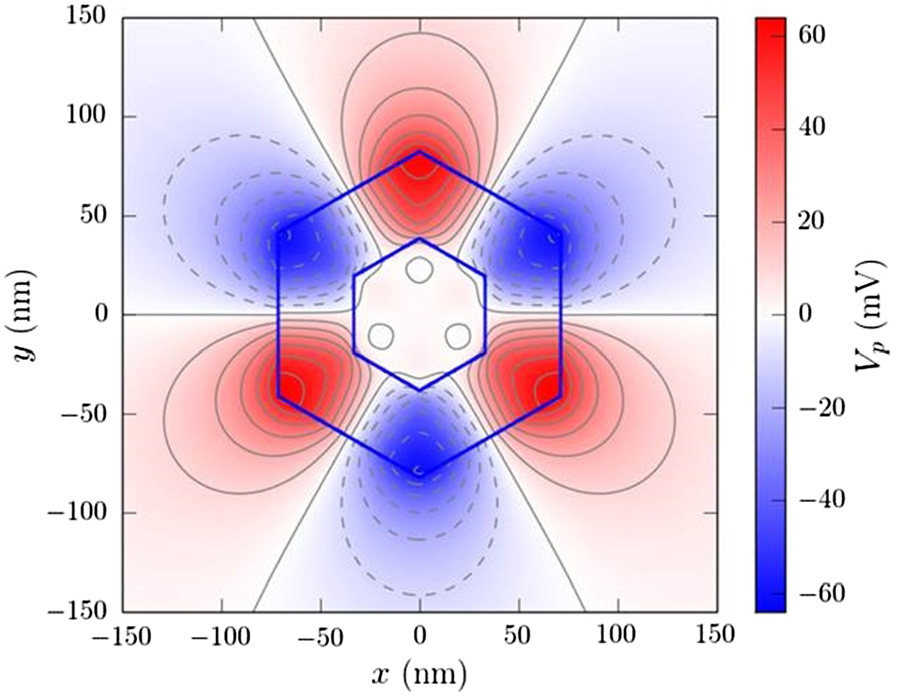
Piezoelectric potential profile for an electron in a cross section of a core-shell nanowire with 70-nm-wide GaAs core and 40-nm-thick In_0.05_Ga_0.95_As shell

As a further motivation for studying the GaAs/InGaAs NW heterostructure in view of PV applications, we plot in Fig. 1 the calculated PZ potential profile over a cross section of a core-shell NW with a 70-nm-wide GaAs core and a 40-nm-thick In_0.05_Ga_0.95_As shell. In this calculation, the structure is relaxed with Keating’s valence force field [14]. Then, the PZ polarization density is computed from the strain tensor *ε*_*ij*_ and Poisson’s equation is solved for the PZ potential. A virtual alloy is assumed in the InGaAs shell (averaging the elastic and piezoelectric constants of InAs and GaAs). Details can be found in [14]. Aside from the axial component of the PZ polarization which can generate significant axial PZ fields, as mentioned above, we show in Fig. 1 that the lateral components of the PZ polarization give rise to the formation of periodic maxima and minima in the potential profile with a clear triangular symmetry. The potential difference between the maxima and minima is already ~100 mV in the given heterostructure, which is substantial enough to ensure that the photo-generated charge carriers will be efficiently separated in different parts of the NW, thus minimizing the carrier recombination losses—a very important parameter in optimizing PV devices.

**Fig. 2:**
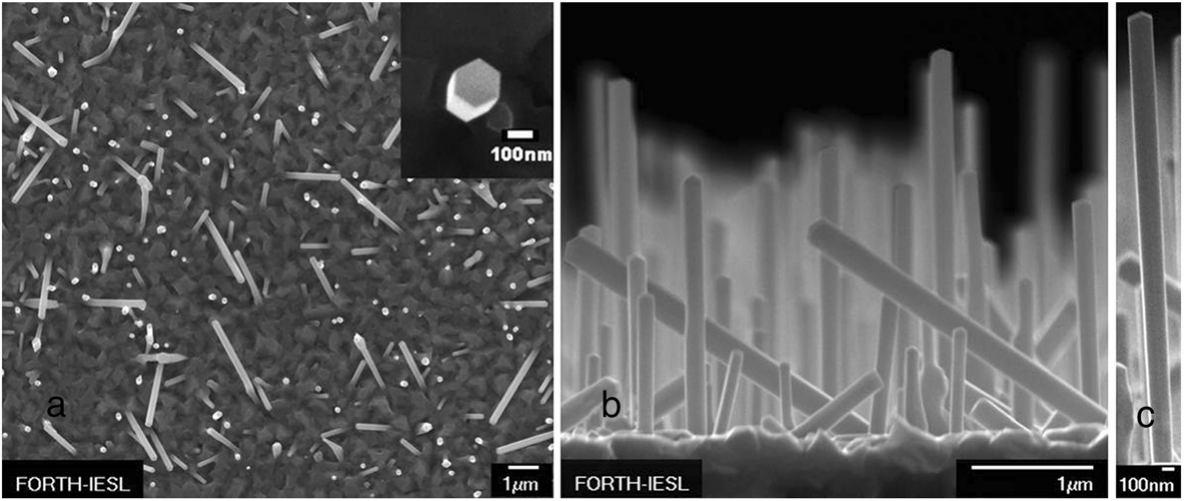
**a** Top view FE-SEM image of core-shell GaAs/InGaAs nanowires with ≈50-nm shell thickness. The *inset* shows the hexagonal morphology with {110} facets. **b** Side view of the same sample. **c** Close-up view illustrating small tapering effect of the core-shell nanowires

Typical FE-SEM images of core-shell GaAs/InGaAs NWs are shown in Fig. 2, where we observe that over 60 % of the NWs are grown along the [111] direction perpendicular to the substrate, while the rest grow along inclined {111} crystallographic orientations, consistent with results reported previously for similar growth conditions [15, 16]. By closer inspection of the SEM images, most of the core-shell NWs display characteristic hexagonal morphology with {110} lateral facets (cf*.* inset of Fig. 2a), as well as a small tapering effect with the NW diameter increasing by 5–10 % along the NW growth axis (cf*.* Fig. 2c). Among the SEM images of the three GaAs core NW reference samples, no significant variation in the NW density and dimensions is observed from sample to sample, signifying a good control of the VLS growth conditions. The average diameter of the GaAs core NWs is 58 nm, the average length is 3 μm, and the density is in the order of 10^8^ nanowires per cm^2^. In the case of GaAs/InGaAs core-shell NWs, an InGaAs shell growth duration between 1 and 40 min results in average InGaAs shell thickness between 1 and 50 nm. On the other hand, the average NW length only increases gradually from 3 to 3.5 μm for InGaAs shell growth duration between 0 (i.e., without shell) and 40 min. This suggests that the growth rates at the NW tips are 5–10 times higher than at the lateral facets. Consequently, an InGaAs region forms around the tip, with a length about 5–10 times the InGaAs shell thickness. The higher growth rates at the NW tip can be attributed to “shadowing” effect and to varying growth rates at the different crystallographic planes of the tip and lateral facets.

Next, we attempt to characterize the *actual* incorporation and distribution of In in the core-shell NWs. Energy-dispersive X-ray (EDX) analysis on a range of relatively large areas (1–10^5^ μm^2^) of the as-grown samples reveal In contents that are in good agreement with the nominal values. For instance, EDX gives an In content of 7.5 % for the sample of Fig. 2, compared with the 9.5 % nominal concentration. However, due to the relatively low NW density, most of the In signal in such EDX scans originates from the polycrystalline InGaAs 2D layer formed at the interface with the Si substrate in between the NWs. In other words, the EDX results may not necessarily represent the true level of In incorporation in the InGaAs NW shell. EDX measurements on single NWs were not conclusive because the In signal was below the detection limit of the system.

**Fig. 3:**
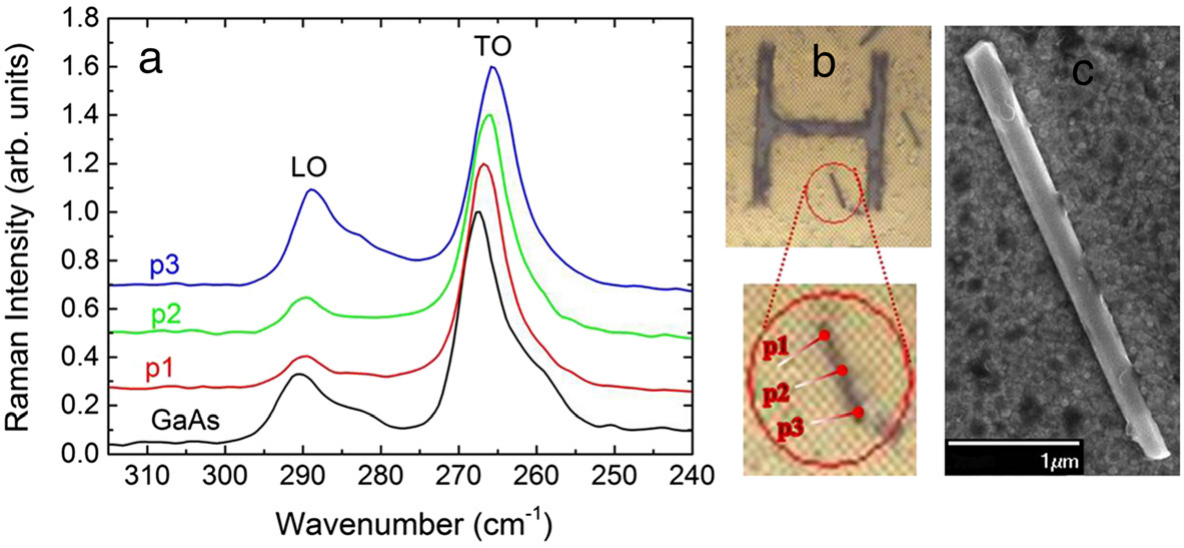
(*color online*) (**a**) Micro-Raman spectra obtained from three different points along a single GaAs/InGaAs core-shell nanowire with ≈50-nm shell thickness. The Raman spectrum of a GaAs (111)B substrate is also plotted as reference. **b** Optical microscope images indicating the three points on the nanowire from which the Raman spectra are obtained. **c** SEM image showing details of the same nanowire in higher resolution

To further investigate the question of In incorporation and distribution in the NWs, micro-Raman measurements were performed on single NWs dispersed on a grid (Fig. 3). In Fig. 3a, typical micro-Raman spectra from a single GaAs/InGaAs core-shell NW are depicted and compared with a GaAs (111)B substrate. The NW spectra are obtained from three different points along the NW axis, as indicated in the optical microscope images of Fig. 3b. Considering the small tapering of the NW, better distinguished in the SEM image of Fig. 3c, point 1 corresponds to the bottom part of the NW, and point 3 to the top part of the NW (“tip”). The In content can be estimated by comparing the positions of the phonon peaks with the relations found in the literature [17] for the GaAs-like longitudinal optical (LO) and transverse optical (TO) phonon modes of In_*x*_Ga_1−*x*_As. In Fig. 3a, both phonon modes seem to shift to lower wave numbers as the NW is scanned from the bottom to the top, suggesting that the In incorporation increases along the growth axis towards the NW tip. For the particular NW in Fig. 3, the In contents deduced from the LO phonon peaks are 1.7, 1.7, and 3 % for points *p*_1_, *p*_2_, and *p*_3_, respectively, while the corresponding values from the TO phonon peaks are 2.7, 4.7, and 6.7 %, respectively. This highlights the fact that the In contents determined by the Raman shifts of the LO phonons are systematically lower than the values obtained from the TO phonons. We interpret this as an effect of strain in the InGaAs shells affecting the TO and LO phonons in opposite directions [18]. To take into account this strain effect, we employ the average value of the In contents, determined by the two phonon modes. Hence, the average In content for the specific NW of Fig. 3 would be 2.2 % at the bottom, 3.2 % in the middle, and 4.8 % towards the top. Similar findings have been found in at least another 15 NWs studied, allowing us to conclude that the In incorporation in the core-shell structure exhibits gradients along the NW axis and is on average significantly smaller than what is nominally expected, which can be attributed to different In incorporation in the various crystallographic planes of the NW structure.

**Fig. 4:**
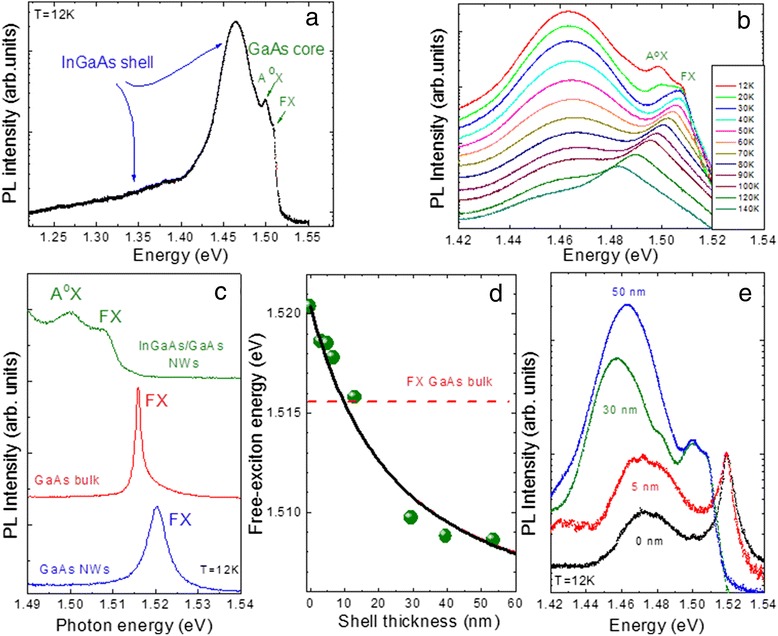
(*color online*) (**a**) PL spectrum at 12 K from a core-shell NW sample with ≈50-nm shell thickness. **b** Temperature-dependent PL measurements from the same core-shell NW sample with ≈50-nm shell thickness. **c** Comparison of low-temperature PL spectra at the GaAs band edge between the core-shell NW sample with ≈50-nm shell thickness, an undoped GaAs bulk sample, and reference GaAs NWs. **d** GaAs core free exciton energy position at 12 K as a function of InGaAs shell thickness. **e** Comparison of low-temperature PL spectra for core-shell NW samples with varying shell thicknesses

The above finding of smaller In incorporation in the NWs appears to agree with the emission properties of the as-grown samples. In Fig. 4a, we show a typical low-temperature PL spectrum from a core-shell NW sample with ≈50-nm shell thickness. Focusing first on the high energy side, we distinguish two GaAs core-related peaks at 1.509 and 1.500 eV, which are assigned as emission from GaAs-free-like excitons (FX) and excitons bound to neutral acceptors (A^0^X), respectively [19]. This assignment is justified by their temperature dependence, as shown in Fig. 4b, where we observe a rapid ionization of the A^0^X peak with temperature, while the FX line persists and dominates the whole spectrum. In Fig. 4c, we compare the FX emission peaks of the core-shell NWs with ≈50-nm shell thickness, an undoped GaAs bulk sample, and the reference GaAs NWs. A first observation is that the FX peak of the reference GaAs NWs, situated at 1.520 eV, is blueshifted by as much as 4.5 meV with respect to the FX position of bulk GaAs. This blueshift is attributed to quantum confinement effects as the NW diameter decreases [20]. On the other hand, the FX peak of the core-shell NW sample is characteristically redshifted with respect to the reference GaAs NWs, and as shown in Fig. 4d, the redshift increases with the InGaAs shell thickness, clearly suggesting that it is caused by the strain imposed to the core by the shell layer. Similar redshifts have been observed previously in other systems including GaAs/AlGaAs NWs [12]. It can be shown [21] that the strain-induced variation of the gap in the core layer can be written as

$$ {E}_X={E}_X^0-\left({a}_c+{a}_{\upsilon}\right)\cdot \frac{c_{11}-{c}_{12}+6{c}_{44}}{c_{11}+{c}_{12}+2{c}_{44}}\cdot f\left(1-\eta \right)-\frac{d}{\sqrt{3}}\cdot \frac{c_{11}+2{c}_{12}}{c_{11}+{c}_{12}+2{c}_{44}}\cdot f\left(1-\eta \right) $$

where, aside from the elastic constants and deformation potentials of the core material, *f* is the lattice mismatch between GaAs and InGaAs and (1 − *η*) is the cross-sectional area ratio between the InGaAs shell and the full core-shell structure, including the GaAs cap layer. The dependence on the shell thickness is contained in this area ratio. Using GaAs parameters [22], the average core and core-shell NW diameters and a 3-nm GaAs cap layer thickness, we can reproduce the data as represented by the solid line in Fig. 4d, assuming a lattice mismatch of ~0.1 %. This value corresponds to an average In concentration in the shell layer of merely ~1.5 %, in good agreement with the micro-Raman results.

**Fig. 5:**
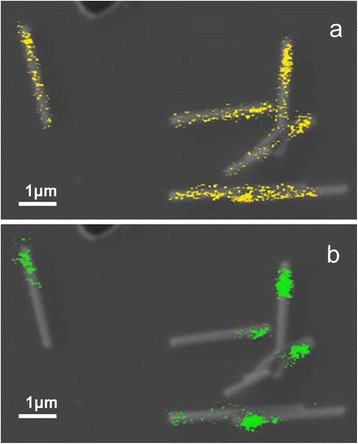
(*color online*) Typical CL mappings at T = 5 K from a bundle of dispersed nanowires on Si, spectrally filtered at the indicated energies of (**a**) 1.508 ± 0.005 eV (*top*), where a relatively uniform emission attributed to the GaAs core is observed along the nanowire, and (**b**) 1.475 ± 0.005 eV (*bottom*) where strong emission from InGaAs is obtained at the nanowire tip. The *scale bar* is 1 μm

Returning to the full PL spectrum of the core-shell NWs in Fig. 4a, the InGaAs shell gives rise to a broad emission spectrum from about 1.48 eV down to 1.23 eV. The intense PL peak around 1.46–1.47 eV, which is present in all core-shell NW samples with shell thicknesses above 10 nm, cannot be attributed to donor-acceptor-type recombination [23] in the GaAs core for two reasons: firstly, because it dramatically acquires strength as the shell thickness increases, as visible in Fig. 4e, and secondly, based on the CL measurements that will be discussed next. In Fig. 5, typical CL mappings spectrally filtered at two different energies are shown from an ensemble of dispersed core-shell NWs on a grid. While the GaAs core emission at 1.508 ± 0.005 eV (top) seems to originate from nearly the whole length of the NW, the emission at 1.475 ± 0.005 eV (bottom) is localized on one end of the NW which, based on the slight tapering effect, corresponds systematically to the NW tip. As explained above, the NW tip consists of an InGaAs region of several hundreds of nanometers in length, without the GaAs core; based on its emission energy [24] and neglecting any confinement effect, the In content at the NW tip can be estimated around 4 %, again in good agreement with the micro-Raman results.

**Fig. 6:**
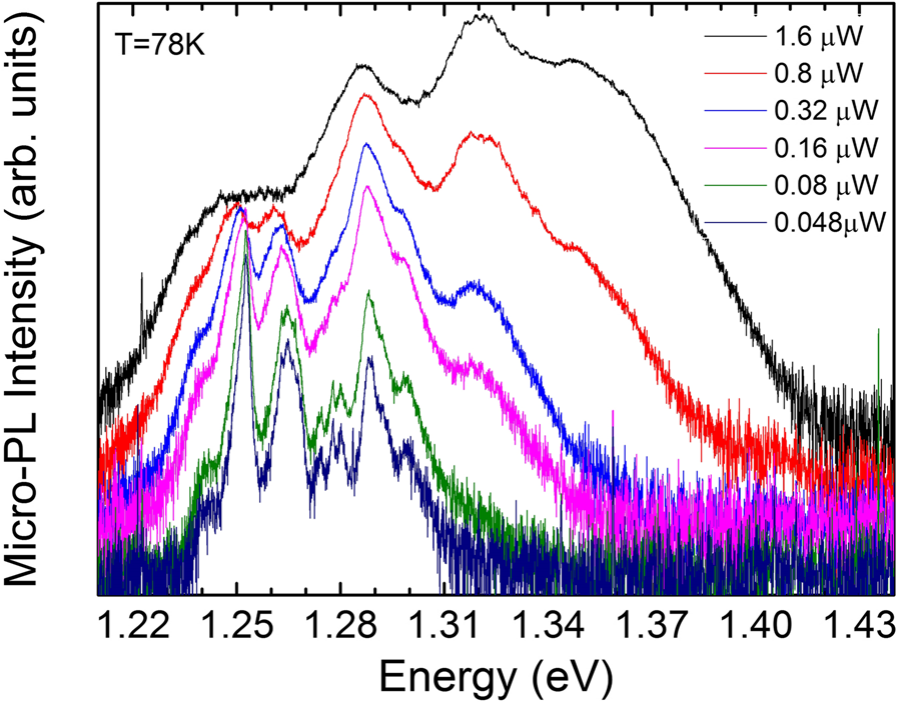
(*color online*) Power-dependent micro-PL spectra at 80 K from a single nanowire, where a quantum dot-like emission signature becomes evident at low excitation powers

To understand the nature of the InGaAs-related emission at energies below 1.4 eV, micro-PL measurements are performed on single NWs. Typical micro-PL spectra at 80 K as a function of power are shown in Fig. 6, where at low powers “sharp” dot-like emission features are observed at energies as low as 1.25 eV, strongly suggesting the existence of In-rich enclosures. From the energy position, we can estimate that the local In content should be larger than 30 %. With increasing excitation power, a spectacular power-dependent evolution of the spectra is observed, with the emission spectra blueshifting due to successive state filling effects. The micro-PL signature differs not only from one NW to another but also between different parts of the same NW, suggesting the formation of In-rich enclosures in various parts of the NW. At this stage, it is not possible to distinguish whether the In-rich enclosures are in the form of channels along the <112> direction [25, 26], or in the form of In shell aggregates in a quantum dot-like structure [27]. Ongoing high-resolution TEM measurements and time-resolved micro-PL will shed more light on the structural properties of the InGaAs shell and the morphology of the presumed In-rich enclosures mentioned above.

## Conclusions

Strained core-shell GaAs/In_*x*_Ga_1−*x*_As nanowires with nominal In content between 2 and 9.5 % have been successfully grown on Si (111) substrates, with the majority of the nanowires growing vertical to the substrate. The as-grown nanowires are 3–3.5 μm long, have a density of about 10^8^ cm^−2^, a core diameter of ~60 nm, and an InGaAs shell thickness ranging from 1 to 50 nm, depending on the shell growth duration. By applying a range of optical and structural characterization techniques, we conclude that the In incorporation in the nanowires is on average significantly smaller than the nominal In content estimated from 2D InGaAs growth calibrations. The In content also exhibits a gradient along the nanowire axis, with higher In content at the tip compared with the lateral facets, which can possibly be attributed to different In incorporation rates in the various crystallographic planes of the nanowire surface. Moreover, the dot-like emission spectra of single nanowires show evidence of the formation of In-rich enclosures with an In content higher than 30 %.
